# Theory to practice: a success in breeding sugarcane variety YZ08–1609 known as the King of Sugar

**DOI:** 10.3389/fpls.2024.1413108

**Published:** 2024-05-14

**Authors:** Qibin Wu, Aomei Li, Peifang Zhao, Hongming Xia, Yuebin Zhang, Youxiong Que

**Affiliations:** ^1^ National Key Laboratory of Tropical Crop Breeding, Institute of Tropical Bioscience and Biotechnology, Chinese Academy of Tropical Agricultural Sciences/Sugarcane Research Institute, Yunnan Academy of Agricultural Sciences, Sanya, China; ^2^ National Key Laboratory of Tropical Crop Breeding, Institute of Tropical Bioscience and Biotechnology, Chinese Academy of Tropical Agricultural Sciences/Sugarcane Research Institute, Yunnan Academy of Agricultural Sciences, Kaiyuan, China; ^3^ Key Laboratory of Sugarcane Biology and Genetic Breeding, Ministry of Agriculture and Rural Affairs, National Engineering Research Center for Sugarcane, College of Agriculture, Fujian Agriculture and Forestry University, Fuzhou, China; ^4^ Key Laboratory of Sugarcane Biotechnology and Genetic Improvement (Guangxi), Ministry of Agriculture and Rural Affairs, Guangxi Academy of Agricultural Sciences, Nanning, China

**Keywords:** sugarcane, hybrid breeding, YZ08–1609, genetic characteristics, biological basis

## Abstract

Sugarcane, a significant cash crop in tropical and subtropical regions, contributes to 80% of sugar production and 40% of bioethanol production in the world. It is a key sugar crop, accounting for 85% of sugar production in China. Developing new varieties with high yield, high sugar, and better stress resistance is crucial for the sustainable growth of sugar industry. Hybrid breeding is the most widely used and effective method, with over 98% of Chinese sugarcane varieties resulting from this approach. Over the past two decades, Chinese breeders have developed the theory of high-heterogeneous composite high-sugar breeding, leading to the successful breeding of the fifth-generation sugarcane varieties. Among them, YZ08–1609, a complex hybrid of *Saccharum* spp., was developed by Sugarcane Research Institute (YSRI) of Yunnan Academy of Agricultural Sciences. The average cane yield of YZ08–1609 was 14.4% higher than ROC22. It is highly resistant to mosaic disease, and highly tolerant to drought stress, but moderately susceptible to smut disease. Notably, YZ08–1609 stands out with a sucrose content of 20.3%, setting an international record, earning the reputation as “King of Sugar”. To summarize experience and inspire breeding, we provided here the detailed insights into the selection of parents, breeding process, and characteristics of YZ08–1609. Besides, the biological mechanisms underlying its high yield and high sugar was excavated at both transcriptional and metabolic levels. The challenges and prospects in breeding sugarcane varieties especially with high sugar were also discussed, offering a foundation for the future development of high-sugar varieties.

## Introduction

Sugarcane (*Saccharum* spp), known for its high photosynthetic efficiency as a C4 plant, is a crucial cash crop in the tropics and subtropics. It accounts for 80% of sugar production and 40% of bioethanol production in the world. In China, sugarcane is a prominent sugar crop, with sucrose contributing to over 85% of sugar production (Lam et al., 2019; Rajput et al., 2021). In recent years, with socio-economic development and the irreversible urbanization, the cost of planting sugarcane has been increasing year by year, but the international sugar price has been consistently low since 2011. Accordingly, Chinese sugar factories have generally suffered great losses, which has seriously affected the motivation of sugar producers, and the sugarcane planting area and sugar production have continued to decline (Li et al., 2017; Ma and Xu, 2013). Besides, over the years, sugarcane breeding has faced challenges mostly due to the accumulation of diseases, aggravating the varietal degeneration. Developing new varieties with high yield, high sugar, and better stress resistance is crucial for the sustainable growth of sugar industry (Lu et al., 2024). It will improve the cane yield and sucrose content of sugarcane, reduce the cost of sugar production, fulfill the demand for good varieties, and thus enrich and rationalize the layout of sugarcane varieties in planting areas (Xia et al., 2018). For sugarcane breeding, methods mostly include sexual hybridization, transgene, molecular marker-assisted selection, and mutagenesis (Chen et al., 2011). Hybrid breeding is the most widely used and effective method, with over 98% of Chinese sugarcane varieties resulting from this approach. At home and abroad, breeding efforts primarily rely on hybridization, which has historically played a significant role in improving sugarcane varieties in different countries (Zhang et al., 2022; Que et al., 2024; Zhang et al., 2024).

In recent years, there has been a shift to dry slopes in Chinese sugarcane cultivation, and from 2000 to 2013, mostly (about 85%) was occupied by ROC series varieties (Liang et al., 2021). This shift has raised concerns about national sugar security and the sustainability of the sugarcane industry. Consequently, there is a pressing need for the selection and promotion of new sugarcane varieties with high yield and high sugar to address these challenges. Over the past two decades, sugarcane scientists have developed the theory of high-heterogeneous composite high-sugar breeding to meet the scientific and technological needs of sugarcane production in China (Zhang et al., 2022, 2024). They have innovated methods for high-sugar breeding to address technical challenges in directional crossbreeding. Additionally, they have devised a high-sugar selection procedure that combines the ideas of parental combination, pedigree evaluation, early generation selection, ideal plant type and multi-dimension assessment, leading to the successful breeding of several new high-yield and high-sugar (the fifth-generation) sugarcane varieties, including but not limited to Yunzhe 08–1609 (YZ08–1609), YZ05–51, Guitang 42 (GT42), GT44, and Liucheng 05–136 (LC05–136) (Zhang et al., 2022, 2024). These varieties now represent over 83.4% of cultivation in Chinese sugarcane producing regions, mainly Guangxi, Yunnan, Guangdong and Hainan provinces. Among them, YZ08–1609, was bred by Sugarcane Research Institute (YSRI) of Yunnan Academy of Agricultural Sciences. It was selected from the cross YZ94–343 × Yuetang 00–236 (YT00–236) made by YSRI, and the fuzz was produced at Yacheng Breeding Station of the Guangdong Sugarcane Research Units. The female parent, YZ94–343, is an advanced clone developed by YSRI, while the male parent, YT00–236, is a cultivar developed by Guangdong Sugarcane Research Units. During the 14th Yunnan regional trial for sugarcane varieties, it stands out with a sucrose content of 20.3%, setting an international record (Xia et al., 2018; Zhao et al., 2019). In order to summarize experience and inspire sugarcane breeding, we provided here detailed insights into the selection of parents, breeding process, and characteristics of YZ08–1609. Besides, the biological mechanisms underlying its high yield and high sugar are then elaborated at both transcriptional and metabolic levels. Finally, challenges and prospects in the selection and breeding of sugarcane varieties were discussed. We hope that it can offer a foundation for the future development of high-sugar varieties.

## History of sugarcane breeding in China

Regarding the development of Chinese sugarcane industry, it has been a process of continuous improvement and renewal of sugarcane varieties. Over the years, China has undergone five rounds of major varietal improvements and renewals (**Table 1**). In the first generation (1955 to 1965), the major planting sugarcane varieties were F134 and F108 form Taiwan and Co419 and Co290 from Indian. The second generation (1965 to 1978) included varieties like YT57–423 and YT63–237 from Guangdong, Chuangtang 61–408 (CT61–408) from Sichuan, and YZ65–225 from Yunnan. The third generation (1978 to 2000) contained GT11 and GT15 from Guangxi, YZ71–388 from Yunnan, and YT86–368 from Guangdong. The fourth generation (2000 to 2013) was mostly occupied by ROC10, ROC20, ROC22, and ROC25 from Taiwan, as well as YT93–159 from Guangdong. Subsequently, after 2013, scientists successfully bred several new high-yield and high-sugar sugarcane varieties, including YZ08–1609, YZ05–51, GT42, GT44, and LC05–136 (the fifth generation). These advancements have significantly propelled the sugarcane industry in China and established the country as a major global sugar producer, making a substantial contribution to sugar supply (Zhao et al., 2022).

**Table 1:** Five generations of major sugarcane varieties in China.

Time period	Generation	Major varieties	Parents	Breeding units
1955–1965	First	F134	Co290 × POJ2878	Taiwan Sugar Research Institute, China
		F108	POJ2725 × F46	Taiwan Sugar Research Institute, China
		Co419	POJ2878 × Co290	Indian Institute of Sugarcane Breeding
		Co290	Co221 × D74	Indian Institute of Sugarcane Breeding
1965–1978	Second	YT57–423	F108 × F134	Guangdong Sugarcane Research Units
		YT63–237	Co419 × CP33–310	Guangdong Sugarcane Research Units
		CT61–408	NCo310 × F134	Sichuan Sugarcane Research Units
		YZ65–225	Co419 × F108	Yunnan Sugarcane Research Units
1978–2000	Third	GT11	PT49–50 × Co419	Guangxi Sugarcane Research Units
		GT15	^*^ HN56–12 × ^*^ NJ59–782	Guangxi Sugarcane Research Units
		YZ71–388	YZ65–225 × ^*^ YC59–818	Yunnan Sugarcane Research Units
		YT86–368	F160 × YT71–210	Guangdong Sugarcane Research Units
2000–2013	Fourth	ROC22	ROC5 × PT69–463	Taiwan Sugar Research Institute, China
		ROC20	^*^ PT69–463 × PT68–2599	Taiwan Sugar Research Institute, China
		ROC10	ROC5 × F152	Taiwan Sugar Research Institute, China
		ROC25	PT79–6048 × PT69–463	Taiwan Sugar Research Institute, China
		YT93–159	^*^ YN73–204 × CP72–1210	Guangdong Sugarcane Research Units
2013-now	Fifth	GT42	ROC22 × GT92–66	Guangxi Sugarcane Research Units
		GT44	ROC1 × GT92–66	Guangxi Sugarcane Research Units
		LC05–136	CP81–1254 × ROC22	Liucheng Sugarcane Research Units
		YZ08–1609	YZ94–343 × YT00–236	Yunnan Sugarcane Research Units
		YZ05–51	YC90–56 × ROC23	Yunnan Sugarcane Research Units

*HN, Huanan; NJ, Neijiang; YC, Yacheng; PT, Pingtdong; YN, Yuenong.

## Parental selection and breeding logic of YZ08–1609

**Figure 1:**
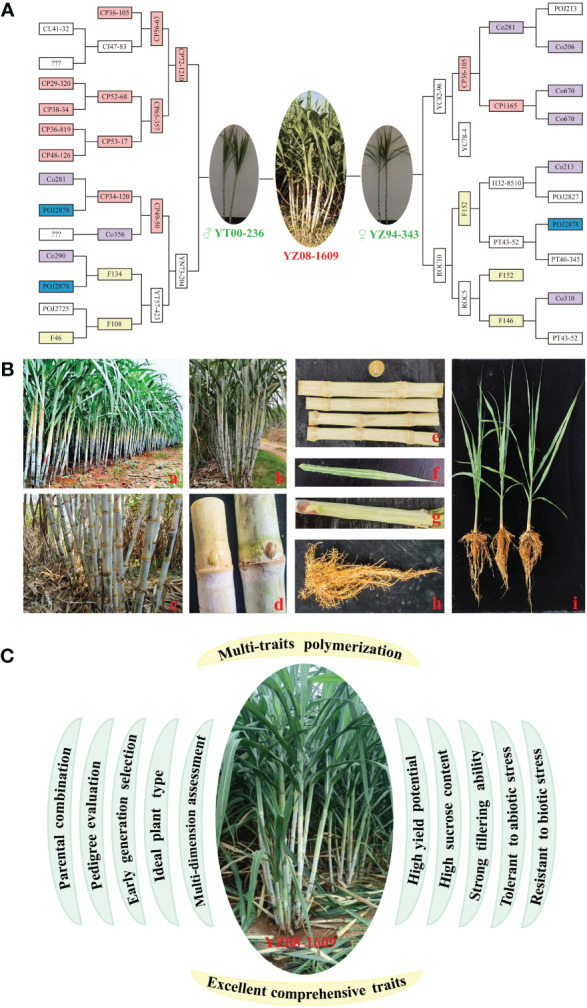
Pedigree, genetic characteristics and breeding procedure for sugarcane variety YZ08–1609. **(A)** A pedigree of YZ08–1609. **(B)** Genetic characteristics of YZ08–1609. a, population; b, monocot; c, stem; d, bud; e, stem section; f, leaf; g, leaf sheath; h, root; i, seedling. **(C)** The breeding procedure for sugarcane variety YZ08–1609 with excellent comprehensive traits.

Generally, the prevalence or wide promotion of an elite sugarcane variety requires high yield, high sugar, and strong resistance (Zhang et al., 2022; Que et al., 2024). To meet the needs of both planting and processing, breeders must carefully balance yield and sucrose content. As the sugar industry advances, there is a growing demand for sugarcane varieties with high sucrose yield, early maturity, and sustained sucrose production throughout the crushing season. Besides, it is crucial to explore and utilize germplasm resources for resistance to diseases, pests, drought, and cold. To support the sustainable growth of sugarcane industry, it necessitates the continuous creation of new germplasm and the adoption of innovative breeding techniques during the process of genetic improvement (Zhang et al., 2022). In the 1910s, Jeswiet introduced the nobleization breeding for sugarcane, crossing tropical species (*S. officinarum*) with live seedlings from Wilbrink (Heinz, 1987). This approach revolutionized genetic improvement in sugarcane, leading to the development of renowned varieties like POJ2714, POJ2725, POJ2878, and POJ2883. Notably, POJ2878, known for its exceptional cane yield, sugar content, resistance, and adaptability, ever dominated 90% of sugarcane cultivation in Java, earning the honor of “King of Sugarcane” (Chen et al., 2011; Zhang et al., 2022, 2024). It served as a crucial parent for sugarcane cross-breeding worldwide, significantly advancing sugarcane breeding. Additionally, core parents like CP, F, and Co series have also played a pivotal role in enhancing sugarcane varieties in China, contributing to 87.63% varieties (Zhang et al., 2009; Qi et al., 2012). Recently, Chinese sugarcane cultivars like LC05–136, YT93–159, YT00–236, and YZ08–1609 have exhibited impressive high sugar, dependent of materials from CP in the United States (Zhang et al., 2022). YZ08–1609, an exceptionally early-maturing variety derived from crossing YT00–236 and YZ94–345, inherits the pedigree of POJ2878, CP, F, and Co series (**Figure 1A**).

## Genetic characteristics of YZ08–1609

YZ08–1609 possesses several elite traits including neat and robust seedlings, strong growth, high stem formation rate, uniform and medium-large stems, and compact and upright plant shape. There is no empty heart. The internodes are conical and buds are pentagonal that reach the growth zone. For leaf, the posture is arching with medium width and greenish color, while sheaths showing medium anthocyanin. The hairs are few, defoliation is good, and ratooning ability is strong. It is highly resistant to sugarcane mosaic disease, and highly tolerance to drought stress, but moderately susceptible to sugarcane smut disease (**Figure 1B**) (Xia et al., 2018; Zhao et al., 2019; Zhang et al., 2022, 2024). Notably, YZ08–1609 exhibits lower resistance to smut disease during the seedling stage and it is prone to falling over once it reaches a height of over 2.0 meters. Under optimal temperature conditions, this variety tends to flower easily, potentially impacting the process of sucrose accumulation. This variety is recommended for planting in paddy fields, dams, dry slopes, and terraces, mostly during the spring, autumn, and winter seasons. It is advisable to plant in fields and dams with medium to high fertility levels, as well as good water and fertilizer conditions. Especially, it is known as the “King of Sugar” with a peak sucrose content of 20.3%, which is the strongest show of continued progress in Chinese sugarcane breeding (Zhang et al., 2022, 2024). In 2023, the cultivation of this variety reached a nominal value of 64,233 hectares, accounting for 6.42% of the total cultivated area in China (Zhang et al., 2024).

## Biological basis of high yield and high sugar in YZ08–1609

**Figure 2:**
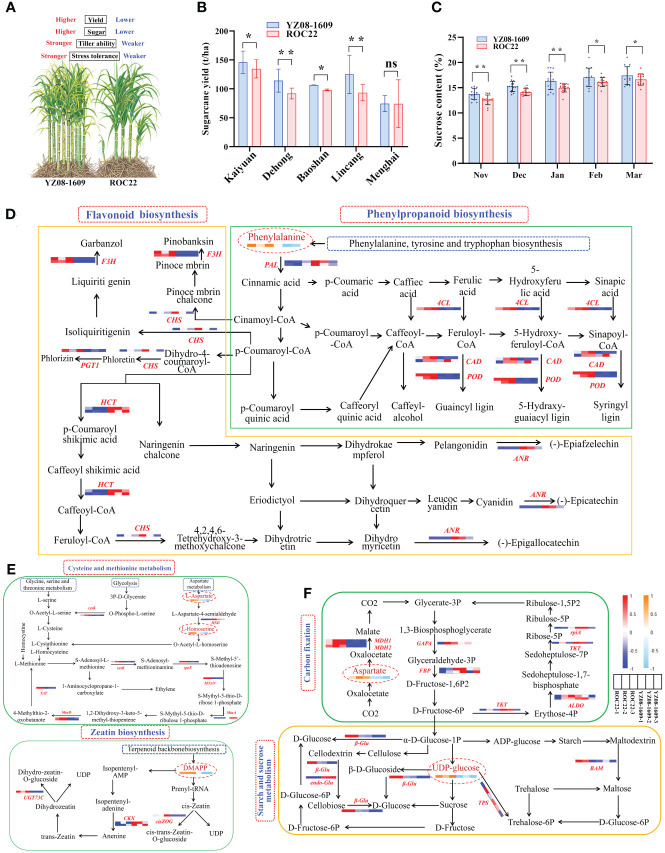
Biological basis of high yield and high sugar in YZ08–1609. **(A)** Key agronomic traits of YZ08–1609. **(B, C)** Cane yield and sucrose content of YZ08–1609 and ROC22 in the 14th Yunnan regional trial (2015–2016, two plant-cane crops and one ratoon crop). The source of data was referenced to Zhao et al. (2019). t/ha represents tons per hectare. Significance (* for *p* < 0.05, ** for *p* < 0.01, and ns for no significance) and standard deviation (SD) were calculated using two-way analysis of variance (ANOVA) followed by Duncan’s new multiple range test of GraphPad Prism 8.0 software. **(D)** Phenylpropanoid and flavonoid biosynthesis. **(E)** Cysteine and methionine metabolism and zeatin biosynthesis. **(F)** Carbon fixation and starch and sucrose metabolism. The heat maps represent the expression of DEGs and DAMs in YZ08–1609 compared to ROC22. Red/orange and blue/sky-blue colors indicate up-regulated and down-regulated genes/metabolites, respectively. PAL, phenylalanine ammonia-lyase; 4CL, 4-coumarate-CoA ligase; CAD, cinnamyl-alcohol dehydrogenase; POD, peroxidase; CHS, chalcone synthase; HCT, shikimate O-hydroxycinnamoyltransferase; F3H, naringenin 3-dioxygenase; PGT1, phlorizin synthase 1; ANR, anthocyanidin reductase; HSD, homoserine dehydrogenase; speE, spermidine synthase; TAT, tyrosine aminotransferase; MTN, 5’-methylthioadenosine nucleosidase; cysK, cysteine synthase; MtnA, methylthioribose-1-phosphate isomerase; MtnD, 1,2-dihydroxy-3-keto-5-methylthiopentene dioxygenase; MDH1/2; malate dehydrogenase 1/2; GAPA, glyceraldehyde-3-phosphate dehydrogenase (NADP+); FBP, fructose-1,6-bisphosphatase I; TKT, transketolase; ALDO, fructose-bisphosphate aldolase; rpiA, ribose 5-phosphate isomerase A; β-Glu, beta-glucosidase; endo-Glu, endoglucanase; TPS, trehalose 6-phosphate synthase. BAM, beta-amylase.

The basis of high yield. Compared to ROC22, YZ08–1609 demonstrated increased yield, higher stress tolerance, and higher tillering ability (**Figure 2A**). During the 14th Yunnan regional trial (2015–2016, two plant-cane crops and one ratoon crop), YZ08–1609 exhibited significantly higher yield than the control ROC22 at four locations (Kaiyuan, Dehong, Baoshan, and Lincang). In addition, the average cane yield of YZ08–1609 was 14.4% higher than ROC22 (**Figure 2B**) (Zhao et al., 2019). It is well known that phenylpropanoid and flavonoid originate from the phenylalanine metabolic pathway, branching into multiple metabolic pathways through the action of key enzymes such as PAL, C4H, 4CL, CCR, CAD, HCT, CCoAOMT, CHS, CHI, F3H, POD, and ANR (Wu et al., 2023). In plants, these pathways effectively regulate the accumulation of ROS in cells, preventing disruption of cellular morphology and metabolic functions. They serve as the primary source of plant defensive secondary metabolites (flavonoids, lignin, and SA), playing a significant role in plant growth, development, and response to environmental stressors (Yadav et al., 2020; Zhang et al., 2020; Dong and Lin, 2021). Interestingly, in the present study, transcriptomics integrated with metabolomics (unpublished) revealed a single differential accumulated metabolite (DAM), L-phenylalanine, and nine differentially expressed genes (DEGs) including *PAL*, *4CL*, *CAD*, *POD*, *CHS*, *HCT*, *F3’H*, *ANR*, and *PGT1* were identified (**Figure 2D**). Previous study demonstrated that sugarcane *ScPAL* gene could enhance the tolerance to cold, drought, salt, H_2_O_2_, as well as the resistance to *S. scitamineum* stress (Song et al., 2013). As reported, *CHS* plays a role in plant growth and development, and in response to biotic and abiotic stress (Glagoleva et al., 2019). It was also unlocked that sugarcane *CHS1* gene could respond positively to the stress by both sugarcane smut fungus and low nitrogen (Wang et al., 2020; Wu et al., 2022).

Abiotic stress tolerance, tillering ability and well development, controlled by cysteine and methionine metabolism as well as zeatin biosynthesis pathways, have a significant impact on sugarcane yield (Liu et al., 2013; Yu et al., 2017). Here in our study, three DAMs (L-Aspartate, L-Homoserine and DMAPP) and 10 DEGs (*HSD*, *speE*, *cysK*, *TAT*, *MTAN*, *MtnA*, *MtnD*, *cisZOG*, *CKX*, and *UGT73C*) were identified to be involved in these pathways (**Figure 2E**). In pepper (*Capsicum annuum* L.), *CaSPDS* played a crucial role in response to cold stress (Zhang et al., 2023). In rice (*Oryza sativa* L.), down-regulation of *OsCKX2* led to the increased number of reproductive organs and ultimately the higher grain yield (Ashikari et al., 2005). Similarly, decreasing the expression of *OscZOG1* could lead to the improvements in panicle branches, total grain number per panicle, and grain weight in rice (Shang et al., 2016). We can thus reasonably deduce that, these DEGs and DAMs involved in phenylpropanoids biosynthesis, flavonoid biosynthesis, cysteine and methionine metabolism, and zeatin biosynthesis pathways played critical roles in enhancing sugarcane growth and development, tillering ability, and resistance to biotic and abiotic stress.

The basis of high sugar. Compared to the control ROC22, YZ08–1609 exhibited a significantly higher average sucrose content during the whole crushing season (November to March) (**Figures 2A, C**) (Zhao et al., 2019). Specifically, in January, February and March, the average sucrose content of YZ08–1609 was 16.35%, 17.04% and 17.42%, respectively, which was 1.39%, 0.93% and 0.79% higher than that of ROC22 (**Figure 2C**). Central carbon metabolism, encompassing carbon fixation and starch and sucrose metabolism, provide energy for plant growth and dry matter accumulation (Winkel-Shirley, 2021). Carbon fixation is vital for plant development and biomass accumulation, as it stores energy and acts as a precursor for the synthesis of key biomolecules like starch and sucrose (Ducat and Silver, 2012; Bar-Even, 2017). For instance, overexpression of *GAPA* acted crucial roles in plant growth and development (Wei et al., 2022). Interestingly, cane yield and sucrose content are heavily influenced by various stages in plant growth such as germination, tillering, stalk elongation, and ripening, all of which can be notably impacted by the carbon fixation and starch and sucrose metabolism (Chen et al., 2019, 2021; Yan et al., 2021). Through a combination of transcriptomic and metabolomic, two DAMs (L-Aspartate and UDP-glucose) and 11 DEGs (*GAPA*, *FBP*, *MDH1*, *MDH2*, *rpiA*, *TKT*, *ALDO*, *β-Glu*, *endo-Glu*, *TPS*, and *BAM*) were identified within the pathways for carbon fixation, as well as starch and sucrose metabolism (**Figure 2F**). Taken together, these DEGs and DAMs involved in carbon fixation, as well as starch and sucrose metabolism and thus contributed to the high sugar in YZ08–1609.

## Conclusion and future perspective

According to the International Society of Sugar Cane Technologists (ISSCT), there are 56 countries with sugarcane breeding organizations worldwide. Major sugarcane producing countries like Brazil, Thailand, India, Australia, and the United States have well-established breeding procedure, with each predominantly using self-breeding for their main planting varieties (Chen et al., 2011; Zhang et al., 2022). In Brazil, main cultivated varieties include RB, SP, and CTC series; in Thailand, they include KK, K, LK, and UT series; in India, that is Co series; and in Australia, they contain Q and KQ series; while in the United States, they are CP, HoCP, and L series. However, there are also some countries and regions, with a later start in breeding, rely more on introduced sugarcane varieties (Chen et al., 2011; Zhang et al., 2022). Currently, almost all sugarcane varieties are derived from existing prevalent varieties as parents, resulting in a narrow genetic basis with limited wild kinship. That is, almost all international varieties contain the kinship of POJ series, and in recent years, newly bred varieties in China are mostly the offspring of CP and ROC series. Besides, the combination configuration is random and the penetration of modern technology is limited. As for the breeding process, it still relies heavily on empirical methods, well known as “pulling and matching for parental combination, and bumping for variety selection” (Zhao et al., 2019; Zhang et al., 2022, 2024).

In the present study, we described a success in breeding sugarcane variety YZ08–1609 known as the King of Sugar, from theory to practice. What excited and inspired us most is that, YZ08–1609, with high sugar content reaching 20.3%, was developed through the cross-breeding of YT00–236 and YZ94–345 using the unique high sugar breeding procedure integrating “parental combination, pedigree evaluation, early generation selection, ideal plant type, and multi-dimension assessment” (**Figure 1C**) (Xia et al., 2018; Zhao et al., 2019; Zhang et al., 2022, 2024). From this perspective, the strategy for selecting and breeding high-sugar varieties can be summarized as follows, introducing wild bloodlines, selection of high-combining parents, rapid focusing of excellent combinations in lineage evaluation, accurately placing outstanding lines in early generation, and then the selection of the ideal plant type in the five-nursery system. Collectively, the present study is helpful to consolidate knowledge and spark innovation in the breeding of high-yield and high-sugar sugarcane varieties.

## Data availability statement

The original contributions presented in the study are included in the article/supplementary material. Further inquiries can be directed to the corresponding authors.

## Ethics statement

Written informed consent was obtained from the individual(s) for the publication of any identifiable images or data included in this article.

## Author contributions

QW: Funding acquisition, Formal Analysis, Methodology, Validation, Writing – original draft. AL: Validation, Writing – original draft. PZ: Writing – review & editing, Data curation, Validation. HX: Data curation, Validation, Writing – review & editing. YZ: Conceptualization, Project administration, Resources, Supervision, Writing – review & editing. YQ: Conceptualization, Funding acquisition, Project administration, Resources, Supervision, Writing – review & editing.
